# Endoscopic retrograde cholangiopancreatography in the treatment of intraoperatively demonstrated choledocholithiasis

**DOI:** 10.1308/003588414X13824511650290

**Published:** 2014-01

**Authors:** AP Lynn, G Chong, A Thomson

**Affiliations:** Canberra Hospital, The Australian National University,Australia

**Keywords:** Choledocholithiasis, Endoscopic retrograde cholangiopancreatography, Cholecystectomy, Laparoscopic common bile duct exploration

## Abstract

**INTRODUCTION:**

The aim of this study was to determine the efficacy and complications of postoperative endoscopic retrograde cholangiopancreatography (ERCP) in confirming and treating choledocholithiasis found at intraoperative cholangiography during laparoscopic cholecystectomy.

**METHODS:**

Patients who had undergone ERCP following a cholecystectomy between 2008 and 2011 with an indication of intraoperative cholangiography findings consistent with choledocholithiasis were identified from a prospectively collected database of a single endoscopist. Deep biliary access rate, confirmation of choledocholithiasis, clearance rate of bile duct stones, delay between cholecystectomy and postoperative ERCP, and the complication rates following the procedure were analysed.

**RESULTS:**

The median age of the 41 patients (16 male, 25 female) was 42 years (range: 18–82 years). Sixteen surgeons performed the operations with a median delay of 6 days (range: 1–103 days) between cholecystectomy and postoperative ERCP. Common bile duct access was achieved in 100% of the patients, with ERCP taking a median time of 16 minutes (range: 6–40 minutes). Initial ERCP confirmed the presence of a stone in 30 patients (73%) and successful stone removal occurred in 28 of these 30 patients (93%) during the first ERCP and in the remaining 2 on a subsequent ERCP. Following ERCP, two patients (4.9%) experienced extended hospital stays for four and eight days owing to complications, including one patient (2.4%) with mild acute pancreatitis.

**CONCLUSIONS:**

This study demonstrates that postoperative ERCP is highly effective in both confirming and treating choledocholithiasis. However, there is a significant risk of short-term complications that must be taken into consideration when deciding management.

The prevalence of gallstones in the population is approximately 15%.^1^ Laparoscopic cholecystectomy is the standard treatment for symptomatic patients. It also prevents future complications including cholecystitis, cholangitis and pancreatitis. Of the patients who undergo a cholecystectomy for symptomatic gallstones, 10–18% are found to have common bile duct (CBD) stones (choledocholithiasis).^1^ In most cases, these stones are secondary in that they have migrated from the gallbladder. Primary choledocholithiasis (stones precipitating along the wall of the CBD) is thought to be seen much less commonly. The presence of CBD stones can be confirmed preoperatively, during cholecystectomy or postoperatively. Intraoperative cholangiography (IOC) during cholecystectomy is now standard practice and may demonstrate a filling defect in the CBD, alerting the surgeon to the possible presence of a CBD stone.

The optimal management of choledocholithiasis demonstrated at IOC is controversial and there are several different approaches to removing such stones.^2^ The approach taken is influenced by multiple factors such as the nature of the presentation, surgeon preference and expertise, and availability of appropriate facilities. Surgical options include laparoscopic common bile duct exploration (LCBDE), which can involve dilating the cystic duct and either retrieving the stone using a guidewire basket or flushing it through into the duodenum. The other LCBDE technique is choledochotomy. Alternatively, patients may be referred for postoperative endoscopic retrograde cholangiopancreatography (ERCP) to have the stone extracted. This occurs especially in centres where the required facilities are lacking or where the technique of LCBDE is unfamiliar or unfavourable to operating surgeons. Another recently advocated approach includes the single stage option of a combined laparoscopic and endoscopic approach, whereby ERCP is performed intraoperatively during the cholecystectomy.^3^

Generally, it is recommended that choledocholithiasis diagnosed prior to surgery should be treated preoperatively or intraoperatively while stones demonstrated intraoperatively should be managed during surgery or postoperatively using ERCP.^4^ Laparoscopic removal of CBD stones is a relatively new technique and despite its proven efficacy in the literature, many surgical teams continue to rely on ERCP.^2^ In this regard, a meta-analysis from 2012 failed to demonstrate any differences in outcome between the one-stage (cholecystectomy + LCBDE) and the two-stage (cholecystectomy + ERCP) approach in patients with suspected choledocholithiasis.^5^

Our study concentrated on the group of patients whose indication for postoperative ERCP was suspicion of choledocholithiasis on IOC during laparoscopic cholecystectomy. The aim was to report on the efficacy and complications of postoperative ERCP in this setting.

## Methods

Data were extracted from an existing prospectively compiled Excel® (Microsoft, Redmond, WA, US) worksheet of consecutive ERCPs performed by a single endoscopist (AT) between 2008 and 2011. This operator did on average over 200 ERCPs per year during the study period. All ERCPs were performed at Canberra Hospital, the principal teaching hospital of the Australian National University. Clinical details of all patients in whom the indication for ERCP was the suspicion of bile duct stones at IOC during cholecystectomy were obtained from the worksheet. The data collected were then cross-referenced with medical records accessed at Canberra Hospital and Calvary Hospital (Canberra’s two largest hospitals). The study was granted ethics approval from both the Australian Capital Territory (ACT) Health Human Research Ethics Committee and the Calvary Health Care ACT Human Research Ethics Committee.

The clinical parameters analysed included the success rate of postoperative ERCP in achieving deep biliary access, the number of cases in which the presence of a CBD stone was confirmed, the rate of CBD clearance in those with ERCP evidence of choledocholithiasis, the delay between cholecystectomy and postoperative ERCP, and the complication rates following ERCP. After ERCP, patients were followed proactively with telephone calls by the endoscopist both on the evening of the procedure and the next day. If the patient was an inpatient, either the endoscopist reviewed the patients in person or telephoned the nursing staff supervising the patient both on the evening of the ERCP and the following day. The definition used for the diagnosis of post-ERCP pancreatitis was epigastric pain with the presence of lipase over five times the upper limit of normal.

Not all patients undergoing cholecystectomy who were found to have IOC consistent with choledocholithiasis were referred for ERCP as in some instances the surgeon was able to remove the stone during surgery. The exact number of such cases is not known but it was lower than the number referred for ERCP. Data concerning choledochotomies performed for intraoperatively demonstrated choledocholithiasis were not collected prospectively. Of the referring surgeons, most do not perform choledochotomies and the total number carried out during the study period for the indication of choledocholithiasis was fewer than five.

## Results

Forty-one patients (median age: 42 years, range: 18–82 years, 16 men and 25 women) were identified who had undergone ERCP following abnormal IOC. All patients had had a laparoscopic cholecystectomy to treat symptomatic gallstones. The procedures were performed in various hospitals in the ACT and south-eastern New South Wales. There were 16 surgeons who performed a cholecystectomy on the 41 patients in the study. Two surgeons performed the most with seven cases each; four surgeons performed three to four cases each. The median time delay between cholecystectomy and postoperative ERCP was 6 days (range: 1–103 days). Two patients had an abnormally long delay between the two procedures (85 and 103 days).

There were 12 patients (29%) treated as inpatients and (71%) as outpatients, only one being an emergency case. Access to the CBD with ERCP was achieved in all patients. The median endoscopy time (from the time the duodenoscope breached the cricopharyngeus to the time that it was withdrawn from the patient) for ERCPs was 16 minutes (range: 6–40 minutes). Three patients had had a previous sphincterotomy; a sphincterotomy or extension of sphincterotomy was performed on all patients, with one exception. Pancreatic stents were placed in 10 patients (24%) to prevent pancreatitis following ERCP.

**Figure 1:**
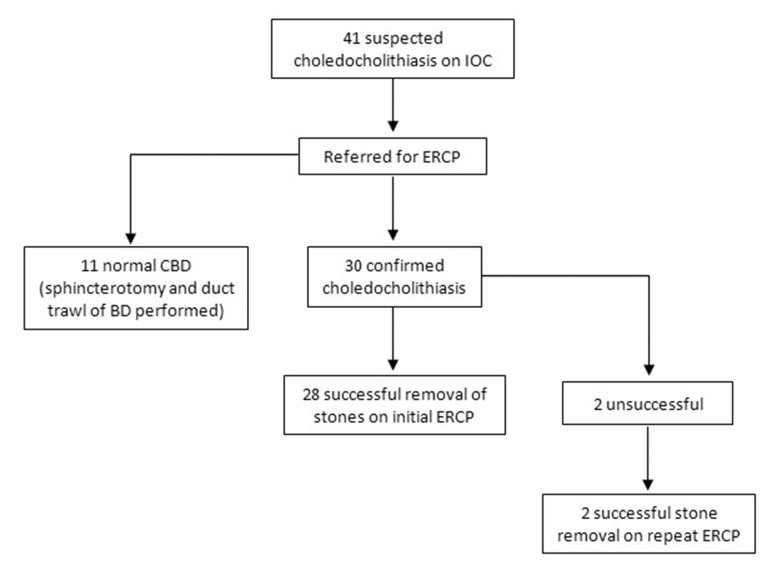
Study profile detailing success of postoperative ERCP in confirming and treating choledocholithiasis demonstrated on intraoperative cholangiography during laparoscopic cholecystectomy

Postoperative ERCP confirmed the presence of a stone in 30 patients (73%). Successful removal of stones with initial ERCP was observed in 28 patients (68%) overall. Two patients required a second ERCP to remove the residual stones and clear the bile duct. The remaining patients had normal cholangiography with no stones seen to come into the duodenum during balloon trawls. Therefore, of the 30 patients in whom a stone was encountered, 28 had their CBD cleared of stones at the first ERCP, giving an ERCP duct clearance success rate of 93% during the first ERCP (Fig 1). The bile duct stones of the other two patients were cleared during a second ERCP with the bile duct being stented during the first procedure.

The operative cholangiography of the 11 patients with normal postoperative ERCPs was available for review in 3 cases. In two of the three, there was a clear filling defect consistent with a stone. In the third, no filling defect was seen in the single available image. In the other eight, either the surgery was done at a small hospital several hundred kilometres from Canberra or hard/digital copies had not been taken. Potentially, some of these patients could have avoided ERCP and its attendant risks.

With regard to complications following ERCP, two patients (4.9%) experienced extended hospital stays due to complications. Of these, one patient (2.4%) was diagnosed with post-ERCP acute pancreatitis, which was non-necrotising and without any long-term sequelae. The other patient was an 82-year-old male inpatient who experienced respiratory distress after ERCP, along with hypoxia and septic showers. He was treated successfully with intravenous antibiotics and fluids. He was diagnosed subsequently with myelodysplastic syndrome. In these 2 patients, the hospital stay lengths following ERCP were 4 and 8 days respectively.

One patient presented to the emergency department with abdominal pain on the evening of her ERCP but absconded before being assessed. A follow-up telephone call confirmed her to be pain free the next day. A fourth patient, a 32-year-old woman with a history of pseudoseizures, experienced a short seizure following ERCP. However, this resolved promptly and did not impact on her recovery or length of stay. None of the other patients experienced significant complications.

No significant differences in complications were found with respect to patient demographics, referral hospital locations (rural or city), time between cholecystectomy and ERCP or ERCP duration.

## Discussion

This study reported on the clinical results of 41 patients who underwent post-cholecystectomy ERCP to confirm and treat intraoperatively demonstrated choledocholithiasis. ERCP confirmed the presence of choledocholithiasis in 73% of the patients. Some of the 11 patients with normal cholangiography at ERCP may in fact have had stones that were not seen to enter the duodenum during balloon trawling of the duct. Alternatively, the cholangiographic abnormalities seen at IOC may have been false positives due to air bubbles or surrounding oedema or spasm.

In patients with confirmed choledocholithiasis, the success rate of the initial ERCP in duct clearance was 93% (Fig 1). This result is higher than reported previously in the literature, possibly owing to recent advances in endoscopic techniques aimed at facilitating CBD clearance. In particular, the use of biliary sphincteroplasty with high pressure balloons before bile duct balloon trawling has now become widespread. In this regard, one patient in the series underwent sphincteroplasty using a 12–15mm CRE™ (Boston Scientific, Natick, MA, US) balloon to remove a stone.

A meta-analysis investigating the various possible surgical and endoscopic approaches to treating choledocholithiasis reported the success rate of duct clearance on the initial postoperative ERCP to be 75%.^1^ There were only two studies in the meta-analysis that compared laparoscopic cholecystectomy plus intraoperative stone removal with laparoscopic cholecystectomy followed by postoperative ERCP. Analysis of these two studies concluded that ERCP performed postoperatively to remove CBD stones was as effective as surgical removal during cholecystectomy, with similar morbidity rates.^6,7^ However, the length of hospital stay in one of the studies was significantly longer in the postoperative ERCP group.^6^ This study, however, only included patients with bile ducts of over 7mm in diameter and therefore investigated a select group of patients. Performing a choledochotomy is likely to be more difficult technically and less successful in patients with more narrow bile ducts.

The much older study by Rhodes *et al*, on the other hand, included all patients in whom evidence for choledocholithiasis was found at cholecystectomy.^7^ Interestingly, in that study, preoperative ERCP was avoided if at all possible – a practice that is not universal. Although there is evidence that conversion to open cholecystectomy is more likely if a preoperative ERCP has been performed,^8^ preoperative ERCP followed by cholecystectomy is an accepted and widely performed treatment option for suspected choledocholithiasis with an in situ gallbladder.^5^ Another approach to intraoperatively demonstrated choledocholithiasis is to place a bile duct stent during surgery. This makes subsequent biliary cannulation easier.

While effective in treating choledocholithiasis, LCBDE is a relatively new technique that is very dependent on the skill of the surgeon and the nature of the patient’s anatomy. The procedure may be demanding technically, and its success relies on the availability of specific resources and equipment, which may be unavailable in certain centres. There is also significant risk as evidenced by a consecutive series of 56 intraoperative choledochotomies from the UK.^9^ The success rate for clearance of the duct was 93% but 11% experienced major morbidity. Half of these required repeat laparoscopy and one patient (2%) required conversion to open surgery.

Despite its proven efficacy, ERCP also has complications and can lead to patient morbidity. Pancreatitis represents the most common serious complication of ERCP.^10^ In our study, one patient (2.4%) developed acute pancreatitis following ERCP. The rate of post-ERCP pancreatitis varies substantially in the literature. A meta-analysis of 21 prospective studies calculated an incidence of 3.75% with a range of 1.6–15.7%.^11^ Two large scale prospective multicentre studies analysing risk factors for pancreatitis following ERCP reported rates of pancreatitis to be 6.7% and 15.1%.^12,13^ A more recent study by Kostrzewska *et al* reported 1.09% of patients experiencing acute pancreatitis after ERCP.^14^ Most of the cases in these studies were mild to moderate, with severe pancreatitis being rare.

Consequently, each strategy has its risks and benefits. The current study highlights that technical improvements in ERCP have occurred in concert with those of surgery. The access rate of 100% and the absence of long-term morbidity are reassuring for the surgeon, who can be confident that there is a safety net if a laparoscopic choledochotomy for stones seen at IOC is, for whatever reason, inadvisable. In addition, the short average ERCP procedure time (16 minutes) makes for low anaesthetic risk. Most of the ERCPs were performed within a week of the cholecystectomy. It is doubtful whether this would have occurred in the era of open cholecystectomies as the postoperative recovery (in particular, the respiratory compromise and the large abdominal wound) made the performance of another procedure within a few days much more hazardous than is the case in the laparoscopic era.

In this vein, one patient in the study, an 82-year-old man, experienced respiratory distress and hypoxia following ERCP, and was diagnosed with myelodysplastic syndrome on discharge. This incident was most likely due to the patient being elderly and having a previously undiagnosed haematological condition. Although major cardiopulmonary complications are rare, studies have shown that they are more commonly seen in patients aged 65 years and older.^15^ Other complications following ERCP that were not observed in this study include haemorrhage, perforation and infection (cholangitis).^10,11^ It can be seen from the literature that there is a large variation in the rates of complications following ERCP. This reflects the differences that exist in the clinical setting such as patient demographics and pre-existing co-morbidities, the complexity of the procedure and the expertise of the endoscopist in performing ERCP.

The strength of the current study is that it was conducted in a demographically isolated major teaching hospital, from which the nearest similar centre (and ERCP service) is over 200km away. Consequently, there is a lack of referral bias compared with previous studies, which have tended to come from areas where patient catchment areas are less distinct. In addition, the good results highlight the benefits of centralisation of expertise with respect to ERCP.

The limitations of this study are its retrospective nature, the relatively small number of cases and the fact that only one endoscopist was involved. The data for deep biliary access and complications following ERCP, however, were collected prospectively and very fastidiously, with contact being made with patients or their carers on the same day of the ERCP and the next day. A much larger sample size with ERCPs being performed by several endoscopists might nevertheless elucidate better the efficacy and complications of ERCP in this context.

## Conclusions

In the laparoscopic era, a uniform approach to the management of intraoperatively suspected choledocholithiasis is lacking. This may never be achieved as the optimal pathway for management is variable and dependent on numerous factors such as the patient’s clinical history, available facilities and the surgeon’s expertise. Postoperative ERCP continues to be used widely in many hospitals. The data from this study support results from previous trials that have shown ERCP to be effective in diagnosing and treating choledocholithiasis. However, our study also shows that ERCP is associated with a modest rate of significant complications. Despite advances in laparoscopic surgery, postoperative ERCP continues to have a role in patients with intraoperatively demonstrated choledocholithiasis.
